# THE IMPACT OF THE OLD AGE PENSION EXPANSION ON BLOOD PRESSURE AMONG OLDER MEN IN RURAL SOUTH AFRICA

**DOI:** 10.1093/geroni/igac059.2894

**Published:** 2022-12-20

**Authors:** Haeyoon Chang, Janet Jock, Molly Rosenberg, Lindsay Kobayashi

**Affiliations:** University of Michigan-Ann Arbor/School of Public Health, Ann Arbor, Michigan, United States; O’Neill School of Public and Environmental Affairs, Bloomington, Indiana, United States; Indiana University/School of Public Health, Bloomington, Indiana, United States; University of Michigan, Ann Arbor, Michigan, United States

## Abstract

The impact of improved socioeconomic welfare after retirement on cardiovascular health among older men in low-income settings is unknown. Using natural experiment, we investigated the impact of eligibility for additional Old Age Pension (OAP) income on systolic blood pressure (SBP) among older men in rural South Africa using data from 1,208 men aged ≥60 in the population-representative “Health and Aging in Africa: A Longitudinal Study of an INDEPTH Community in Rural South Africa” (HAALSI) cohort. The South African Old Age Pension (OAP) incrementally expanded age eligibility for men from age 65 prior to 2008 to age 60 in 2010. This expansion provided exogenous variation in pension income among men, based on their year of birth. We estimated predicted SBP among the birth cohorts of men who were eligible for one through five extra years of OAP income, using a multivariable linear regression model estimated in those without access to extra pension income (over age 65 when the expansion rolled out) based on covariates. We then estimated mean SBP difference scores among men in each of these five birth cohorts, based on their observed SBP minus predicted SBP. Men in OAP expansion birth cohorts had lower SBP than those who were not exposed to extra pension, although the differences were not statistically significant (5-years estimate = -2.735 (p=0.353) vs. 1-year estimate = 1.633 (p=0.531)). This trend suggests a possible long-term benefit of blood pressure control with greater cumulative pension income among men living in rural, low-income settings.

